# Seizure Detection and Network Dynamics of Generalized Convulsive Seizures: Towards Rational Designing of Closed-Loop Neuromodulation

**DOI:** 10.1155/2017/9606213

**Published:** 2017-12-13

**Authors:** Puneet Dheer, Ganne Chaitanya, Diana Pizarro, Rosana Esteller, Kaushik Majumdar, Sandipan Pati

**Affiliations:** ^1^Systems Science and Informatics Unit, Indian Statistical Institute, Kolkata, India; ^2^Department of Clinical Neurosciences, NIMHANS, Bangalore 560029, India; ^3^Department of Neurology, University of Alabama at Birmingham, Birmingham, AL, USA

## Abstract

**Objective:**

Studies have demonstrated the utility of closed-loop neuromodulation in treating focal onset seizures. There is an utmost need of neurostimulation therapy for generalized tonic-clonic seizures. The study goals are to map the thalamocortical network dynamics during the generalized convulsive seizures and identify targets for reliable seizure detection.

**Methods:**

Local field potentials were recorded from bilateral cortex, hippocampi, and centromedian thalami in Sprague-Dawley rats. Pentylenetetrazol was used to induce multiple convulsive seizures. The performances of two automated seizure detection methods (line length and P-operators) as a function of different cortical and subcortical structures were estimated. Multiple linear correlations-Granger's Causality was used to determine the effective connectivity.

**Results:**

Of the 29 generalized tonic-clonic seizures analyzed, line length detected 100% of seizures in all the channels while the P-operator detected only 35% of seizures. The detection latencies were shortest in the thalamus in comparison to the cortex. There was a decrease in amplitude correlation within the thalamocortical network during the seizure, and flow of information was decreased from thalamus to hippocampal-parietal nodes.

**Significance:**

The preclinical study confirms thalamus as a superior target for automated detection of generalized seizures and modulation of synchrony to increase coupling may be a strategy to abate seizures.

## 1. Introduction

Among the different seizure subtypes, generalized tonic-clonic seizures are most disabling due to loss of consciousness, the potential to cause physical injuries, and cardiorespiratory compromise including SUDEP (Sudden Unexpected Death in Epilepsy) [1, 2]. Generalized tonic-clonic seizures can occur in primary generalized epilepsy (now termed as Genetic Generalized Epilepsy) or in partial epilepsy where the seizures are secondarily generalized. Despite therapeutic advances in the treatment of epilepsy, the outcome of primary generalized epilepsy remains suboptimal. When antiseizure medication fails to control seizures, in partial epilepsy resective surgery can potentially cure, but the therapeutic options in generalized epilepsy are limited. Recently an FDA approved closed-loop responsive neurostimulation therapy (NeuroPace) was proven to be beneficial in interrupting seizures including convulsive seizures of partial onset [3]. We wondered if such a treatment paradigm can be extended to control primarily generalized seizures.

Experimental models of generalized epilepsy and functional imaging studies in human suggest that generalized seizures are initiated in cortical nodes, mainly frontal and parietal, before they entrain the thalamus in a bidirectional ictal network [4–7]. Maintenance of the integrity of the reciprocal thalamocortical reverberatory loop is necessary for sustaining ictogenesis. Therefore to design a physiology-based, rational closed-loop stimulation paradigm would require an understanding of the thalamocortical network dynamics during seizure evolution and identifying targets within the thalamocortical loop for reliable seizure detection. To address these critical knowledge gaps, we have performed a preclinical study with the focus on identifying detection metrics that can trigger feedback stimulation effectively to abate generalized convulsive seizures. Specifically, the study aims are to (a) evaluate the performance of automated seizure detectors (line length and P-operator) as a function of different corticosubcortical targets and (b) map the peri-ictal information flow dynamics involving the corticosubcortical network in a validated rodent model of generalized convulsive seizures [8, 9]. The correlation or synchronization of neural activity within seizure-generating sites is implicated in seizure genesis, and desynchronization is a potential mechanism proposed in the stimulation induced suppression of seizures [10–12]. Abnormal synchrony in the corticosubcortical network has also been reported in generalized spike-wave seizures [13, 14]. Therefore, based on these studies, we speculate synchrony as a potential target for rational brain stimulation paradigm. The significance and role of synchrony are likely to depend on the extent of the coupling of interconnected neurons. Coupling measures are used to establish relationship between two EEG signals from different channels of single brain region or two brain regions. Among different methods to analyze coupling, Granger's Causality is one popular method to analyze the coupling strength and direction or information flow of EEG signals between two electrodes in different brain regions. Therefore, using stepwise multiple linear correlations-Granger's Causality analysis, here we explore the spatiotemporal coupling within and between the corticosubcortical networks during seizure evolution. We hypothesize that the causal sources of generalized seizures are spatially distributed within the thalamocortical network and are abnormally correlated in the time domain during seizure evolution. Furthermore, as ictogenesis of generalized seizure involves rapid recruitment of widespread corticosubcortical networks [1], we anticipate that the performance of automated seizure detectors as a function of anatomical targets will be invariant.

Pentylenetetrazol (PTZ), a GABA-A receptor antagonist, is the most commonly used proconvulsant to induce spike and wave, myoclonus, and generalized tonic-clonic seizures [9]. The tonic-clonic seizures induced by PTZ are believed to represent generalized seizures. Unlike genetic models of generalized epilepsy where spike and wave absence seizures are predominant [15, 16], in the PTZ model after administration of the chemical, multiple generalized convulsive seizures, often in clusters, are induced. Since the goal of our study is to define stimulation metrics against generalized tonic-clonic seizures, we felt that the PTZ model suits better than genetic models for our study aims.

## 2. Methods

### 2.1. Animals

Experiments were performed on male Sprague-Dawley rats weighing 400–550 g that were obtained from Charles River Laboratories, MA, USA. Animals were housed in pairs with food and water ad libitum and kept in a 12 h light/dark cycle. All animal experimentations were approved by the Institutional Animal Care and Use Committee. All efforts were made to minimize the animal suffering and reduce the number of animals used in the experiments.

### 2.2. Surgery for Recording Electrodes

**Figure 1:**
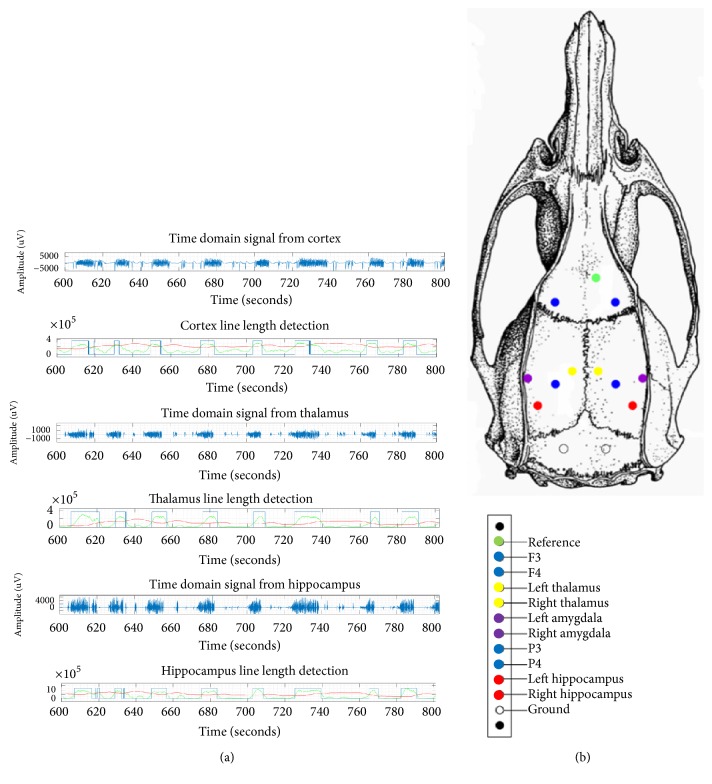
(a) Field potential recordings from the somatosensory (frontal) cortex, centromedian thalamus, and dentate gyrus of the hippocampus during convulsive seizures. Seizures were detected from corticosubcortical structures using line length. (b) Anatomical targets for implantation of depth electrodes.

All procedures are performed using sterile techniques. Rats were anesthetized by inhalation of 5% isoflurane in an induction chamber. The periosteum was cleared from the cranium, and the exposed skull was cleaned with hydrogen peroxide (3%). Once the skull surface was dry, using bregma as the reference location, eight 0.25 mm craniotomies were performed with a stereotaxic drill for placement of monopolar depth electrodes that were custom-made from 175 *μ*m tungsten microwire (California Fine Wire). Four cortical depth electrodes were placed targeting orbitofrontal and somatosensory cortex: AP: 2.0 mm, lateral: ±3.0, and depth: 1 mm; AP: −4.0 mm, lateral: ±3.0, and depth: 1.0 mm. Two hippocampal depth electrodes targeting dentate gyrus (coordinates: AP: −5.6 mm, lateral: ±4.5, and depth: 5.0 mm) and two additional depth electrodes targeting centromedial thalamic nuclei (coordinates: AP: −2.5 mm, lateral: ±1.5, and depth: 5.9 mm at a 10° lateral angle) were placed (Figure 1(b)) [17, 18]. A D-sub connector (pinsout) was then soldered to the recording electrode wires. The connector's ground wire was exposed to the muscle tissue down the back of the neck. The entire head piece was then adhered to the skull using dental acrylic. A day following surgery, the rat was placed in a customized cage, and the head piece was connected to the video-EEG system (Natus Quantum EEG system™).

### 2.3. Seizure Induction and Electrophysiological Recording

Following a 7-day recovery period, seizures were induced by intraperitoneal injection of a single dose of PTZ (130 mg/kg) [19]. Seizures were induced within 20 minutes after administration of the proconvulsant. The recording was continued for 5 hours that allowed recording of multiple seizures. The video EEG was sampled at 1024 Hz.

### 2.4. Defining Onset of Generalized Seizures

Seizures were defined as abnormal electrographic activities lasting more than 10 seconds and associated with relatively high frequency and amplitude [19]. The local field potential (LFP) changes that were associated with generalized tonic-clonic seizures were continuous high frequency, high amplitude spike waves that were present bilaterally on all recorded channels. At times, these ictal electrographic changes transitioned to low-amplitude high-frequency activity before evolving into rhythmic high amplitude spikes during the clonic phase of the seizures. Seizure onset was defined as the earliest occurrence of high frequency, high amplitude spike waves throughout the recording channels that were distinctive from the background activity and that evolved in frequency and morphology. These changes were visually identified and marked as unequivocal electrographic onset (UEO) (Figure 1(a)). The seizure intensity was scored visually using a revised Racine scale that was validated in a previous study [20]. Only seizures that had clear clinical correlates of generalized tonic-clonic activity (stages 5 and 6) were included for analysis.

### 2.5. Automated Seizure Detectors

#### 2.5.1. Line Length

The line length (LL) feature was derived as a simplification of the running fractal dimension of a signal [21]. It measures the length of the signal in a particular window and compares it to a variable threshold. The length of the signal is proportional to the amplitude and frequency of the signal, making this feature highly suitable to sense changes in amplitude and/or frequency that typically occur during seizures.

#### 2.5.2. P-Operator

Clinical analysis of EEG is done by visual inspection. The visually appealing geometric features are therefore of primary importance in EEG signals. If we visualize a time domain EEG signal as the trajectory of a particle moving in a force field with one degree of freedom, then we will be able to trace the evolving geometry of the signal as the motion of the particle with variable acceleration along the ordinate. If *s*(*t*) is the time domain single channel EEG, then the ordinate at a time *t* is *s*(*t*) and acceleration at *t* is *d*^2^*s*(*t*)/*dt*^2^. The work done to displace the particle by an amount *ds*(*t*) along the ordinate is (*d*^2^*s*(*t*)/*dt*^2^)*ds*(*t*). Time taken to accomplish this work is *dt*. So the rate at which the work was done is *P*(*s*(*t*)) = (*d*^2^*s*(*t*)/*dt*^2^)(*ds*(*t*)/*dt*). In classical mechanics, *P*(*s*(*t*)) is known as the power of the moving particle at the point *t*. This is the power at which the particle is giving the specific shape to its trajectory. In other words, *P*(*s*(*t*)) is the power at which the specific waveform of the signal *s*(*t*) at the point *t* is being created. Note that this *P*(*s*(*t*)) has nothing to do with the spectral power of *s*(*t*). Since during an epileptic seizure distinct waveform changes take place in the EEG signal, *P*(*s*(*t*)) of the background EEG and *P*(*s*(*t*)) of the EEG during a seizure should be quite different. This way, *P*(*s*(*t*)) enhances the contrast between the background EEG and the EEG during the seizure leading to convenient seizure detection by setting a suitable threshold or otherwise. The changes are no surprise because the efficacy of first- and second-order temporal difference in seizure detection has already been well documented [22, 23]. We call *P*(•) = (*d*^2^/*dt*^2^)(*d*/*dt*) the power-operator or simply the P-operator. Custom written codes in Matlab (Mathworks, Natick, MA) were used for automated seizure detection.

### 2.6. Automated Detection of Seizures Offline and ROC Curves

A sweep of different thresholds was used to calculate the receiver operating characteristic (ROC) curves. We used the automated detection algorithms to determine true positives, true negatives, false positives, and false negatives for seizure detection. Visual markings of UEO were considered gold standard (Figure 1(a)). We calculated Sensitivity = TP/(TP + FN), Specificity = TN/(TN + FP), and false positive rate FPR = (1 − Sensitivity) for each threshold to build an ROC curve and determine the optimal threshold for each animal and each channel (cortical, hippocampal, and thalamus).

### 2.7. Stepwise Multiple Linear Correlations-Granger's Causality to Determine Effective Connectivity (EC) in the Periseizure Period

**Figure 2:**
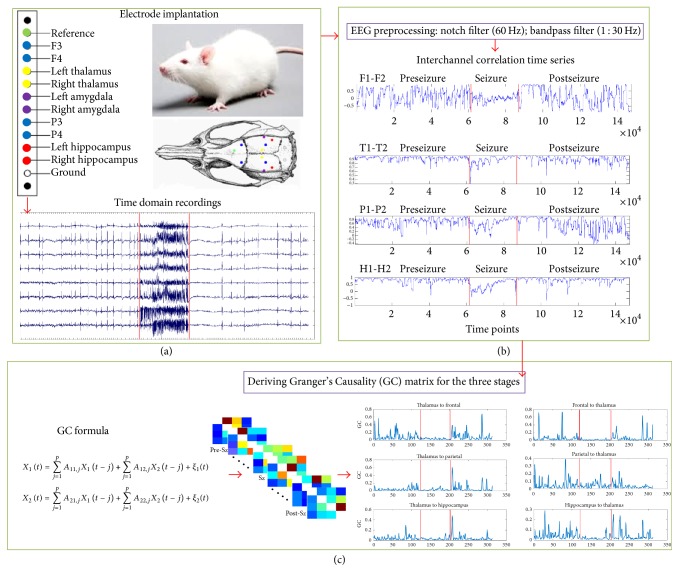
Schematic representation of the analytical pipeline: (a) acquisition of field potential recordings from bilateral depth electrodes, (b) preprocessing of field potentials and analyzing interchannel amplitude correlation, and finally (c) deriving Granger's Causality matrix for seizure and pre- and postseizure states.

The seizure data was visually identified to determine segments preceding the seizure (labeled as PreSz, 1 min), during the seizure (Sz, the length of seizure), and immediately after the seizure (PostSz, 1 min) for analysis of effective connectivity between the channels. Using analysis of time-lagged relationships, effective connectivity is defined as the influence of one neural system exerting over another during an experimental context and therefore moves beyond describing instantaneous connections between brain regions and helps to clarify how brain areas communicate [24]. The eight channels that were used to estimate effective connectivity are placed bilaterally and spatially similar in frontal, centromedian thalami, parietal, and hippocampi. The data (sampled at 1024 Hz) was notch-filtered at 60 Hz and band-pass-filtered using finite impulse response filter between 0.01 and 100 Hz. Initially, multiple linear Pearson's correlations were performed on spatially similar channels using a moving window Pearson Correlation (Ws = 1024 sample points, shifted by 1 sample point) with the pair of frontal, thalami, parietal, and hippocampi. This derived channel was obtained to generate a time series for each of the regions that contained the similar seizure ictal electrical activity between them and not the influence of background activity of the individual regions. Subsequently, the derived frontal, thalamus, parietal, and hippocampal channels were used to analyze effective connectivity (EC) using Granger's Causality (GC), a form of multivariate vector autoregression model determining directed interregional coupling of a collection of time series, measured by one's dependence over the other [25, 26]. GC was calculated across seizure and pre- and postseizure stages using the following parameters: window size of 1024 samples, 50% window overlap, AR order of 10, and number of surrogates of 100 to determine the statistically significant EC (*p* < 0.05, network threshold of 0.8) corresponding to *q* = 0.2 using the type I false discovery rate implementation [27]. The analysis resulted uniformly in 118 temporal windows in both the preseizure and the postseizure periods while the seizure window had variable windows depending on the length of the seizure. A schematic representation of the different pipeline steps is summarized in Figure 2.

### 2.8. Statistical Analysis of the Effective Connectivity Results

Once the GC was calculated for the three stages and across the channels, we tried to establish if the directional connectivity information was dependent on the channels involved or whether it was dependent on the stage of the seizure. Classification of the connectivity across stages and derivations was performed using a data-driven approach combining principle component analysis (PCA) using Varimax rotation and Kaiser Normalization [28, 29]. Finally, the difference in the EC between the three stages was also tested using repeated measures ANOVA with post hoc analysis. A corrected *p* value less than 0.05 was considered significant.

## 3. Results

A total of 29 generalized tonic-clonic seizures (stage 5 and 6 seizure intensity) were analyzed out of 42 seizures recorded from two rats. Thirteen seizures were excluded due to seizure intensity below 5. The duration of the seizure ranged between 14 and 90 seconds. Our first strategy was to compare automated seizure detection against visual detection and plot ROC curves.

### 3.1. Automated Detection of Generalized Convulsive Seizures

**Figure 3:**
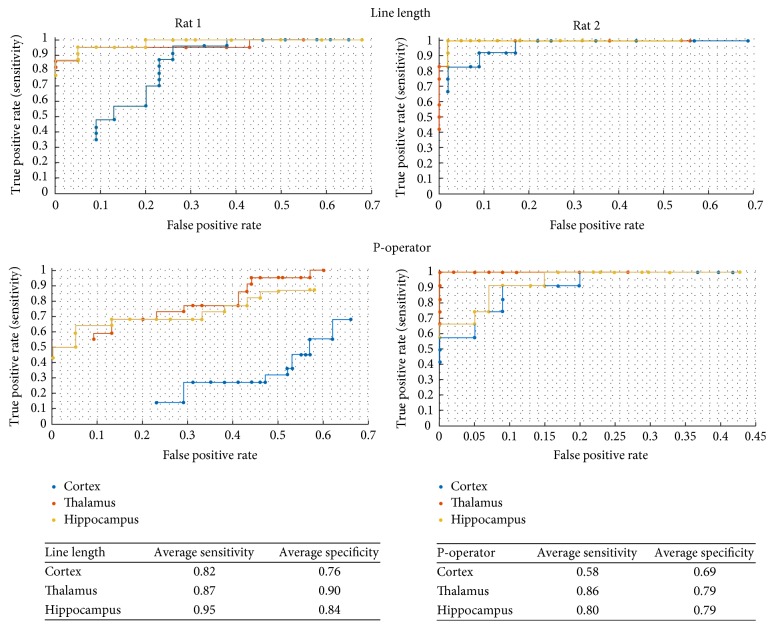
Receiver operating characteristics (ROC) curves to demonstrate the performance of automated seizure detectors (line length and P-operator) as a function of the anatomical targets: frontal cortex, thalamus, and hippocampus.

**Figure 4:**
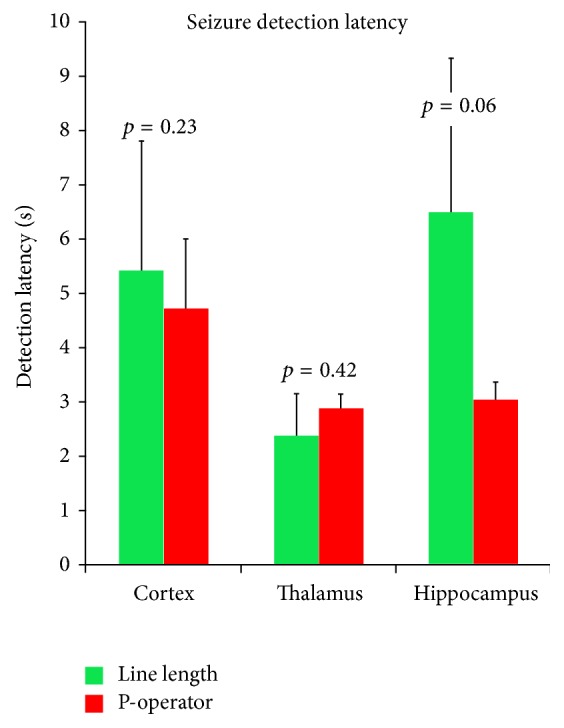
Seizure detection mean latency by automated seizure detectors (line length and P-operator) in cortex, thalamus, and hippocampus.

The line length algorithm detected seizures in all the channels for all the 29 seizures (Figure 1(a)). The mean latency for detection was as follows: frontal cortex, 4.72 ± 6.89 seconds; CM thalamus, 2.87 ± 1.47 seconds; and hippocampus, 3.03 ± 1.84 seconds. The performance of the P-operator was suboptimal as it detected only 35% of seizures (10 each in the thalamus, cortex, and hippocampus) with the latencies varying between them (cortex: 5.41 ± 7.58 seconds; thalamus: 2.36 ± 2.50 seconds; and hippocampus: 6.48 ± 9.02 seconds) (Figure 3). ANOVA was performed to test the difference in seizure detection latency for the two automated detectors (Figure 4). The detection latencies varied as a function of anatomical targets with thalamus superior to frontal cortex for both line length and P-operator.

### 3.2. Stepwise Multiple Linear Correlations-Granger's Causality to Determine Effective Connectivity (EC) in the Periseizure Period

**Figure 5:**
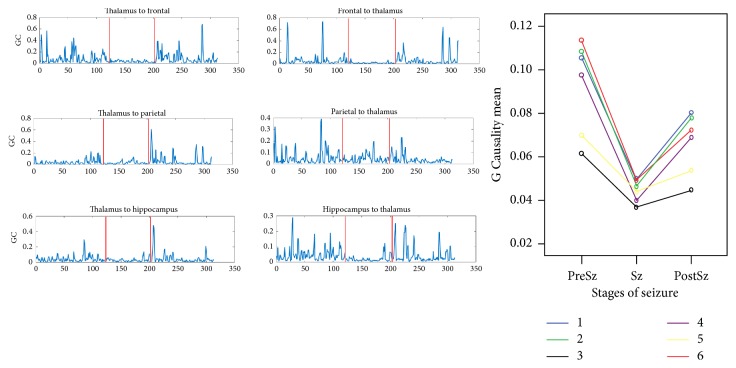
(a) and (b) represent changes in effective connectivity (EC) during seizure (Sz), preseizure (PreSz), and postseizure (PostSz) in the derivations: (1) thalamus to frontal, (2) frontal to thalamus, (3) thalamus to parietal, (4) parietal to thalamus, (5) thalamus to hippocampus, and (6) hippocampus to thalamus. There was a decrease in the EC from the PreSz to the Sz and then a recovery of the EC in the PostSz state that was lower than the PreSz state.

There was a decrease in the linear amplitude correlation among the spatially identical channels (frontal, thalamic, parietal, and hippocampal derivations) during the seizure period. As the baseline (defined in this study as PreSz state) transitioned to seizure, there was a reduction in the coupling strength between all the corticosubcortical channels (Figure 5(a)). In the postseizure state, the coupling between the channels increased but this was not similar to the PreSz state (*p* < 0.0001 for all the derivations) (Figure 5(b)). A data-driven approach classifying the changes in coupling and different seizure states (preSz-Sz-PostSz) showed that the net Granger's Causality could be discriminated into three groups based on the seizures states (Table 1). Repeated measure ANOVA with post hoc analysis was done to evaluate the effective connectivity that had the greatest difference in mean GC between the three stages. The decrease in the flow of information during the seizure was directed from thalamus to hippocampus and from thalamus to parietal nodes. There was no significant difference in the information flow within frontothalamic reciprocal connectivity.

## 4. Discussion

In this study involving mapping of ictal network dynamics in a preclinical model of generalized tonic-clonic seizure, we highlight two pertinent findings that are of translational importance while designing a closed-loop neuromodulation. First, the performance of automated seizure detectors for detecting generalized seizures was variable with subcortical structures (thalami and hippocampi) superior to the cortical structures (frontoparietal). Second, during the generalized tonic-clonic seizure there was a decrease in coupling within the thalamocortical network, and the decrease in information flow was maximum from thalamus to the hippocampus and parietal network.

### 4.1. Subcortical Structures Are Superior Anatomic Targets for Automated Seizure Detection of Generalized Seizures

Performance of an automated detector of seizure onset may be influenced by variability in electrographic signatures of seizures, states of vigilance, site (like scalp versus intracranial EEG), and duration of recording to estimate performance [30–32]. The onset of generalized tonic-clonic seizure involves widespread network incorporating corticosubcortical nodes, and the LFP is stereotyped by high frequency, high amplitude spike waves. Therefore, the results of the ROC curve demonstrating variability in performance between corticosubcortical structures were unanticipated. Our study demonstrated that, even for generalized seizure, the detection latency as a function of anatomical targets is not uniform and that thalamus or hippocampus is a better target for automated seizure detection. Although the differences in seizure detection latency were statistically nonsignificant, from the clinical standpoint a 4-second difference in detection latency can be significant if seizure suppression by intervention is the primary goal of the automated detector. The origin of generalized seizure is debated between cortical focus, thalamus, and thalamocortical network [4, 33–35]. A recent study demonstrated bidirectional control of generalized epilepsy network by switching thalamocortical phasic firing to the tonic state using optogenetic modulation of the thalamus [36]. Our finding of thalamus being superior in automated seizure detection adds to the growing evidence that thalamus may be an attractive target for closed-loop neuromodulation in generalized epilepsy.

### 4.2. Disrupted Connectivity within the Thalamocortical Network during Generalized Tonic-Clonic Seizure

Synchronization (i.e., events occurring at the same time) in epilepsy is conceptually complex, and both decreases and increases in synchrony have been reported with ictogenesis [10]. There are multiple statistical tools to quantify correlation strength and causality among multivariate time series. In this study, we have adopted amplitude correlation (a measure of synchrony) and Granger's Causality to estimate the coupling strength and the direction of information flow. Previous studies with magnetoencephalography (MEG) in patients with generalized seizures have reported fluctuation in synchrony with seizure progression [13, 37]. In generalized spike-wave absence seizure, there was long-range desynchrony at onset followed by local and long-range synchronization as seizure progressed. For generalized motor seizure, the increase in global synchrony (as measured by phase synchrony) was lesser compared to absence seizure. In the present study, there was a decrease in the coupling of field potentials within the thalamocortical reverberatory loop with seizures and the decrease in connectivity was directed from thalamus to hippocampus and parietal network. Mapping the ictal network dynamics might influence the temporospatial selection of stimulation parameters. Although debated, one proposed mechanism by which high frequency (>150 Hz) stimulation suppresses seizures is through desynchrony [11, 38]. If decrease in coupling within the thalamocortical network is the predominant change during ictogenesis of generalized convulsive seizure, one might speculate if increasing coupling by altering frequency (low or high frequency) or phase resetting stimulation can effectively abate seizure [39–41]. Indeed low- and high-frequency stimulation have been shown to decrease seizure activity in several animal models of epilepsy [42–44].

## 5. Study Limitations

The goal of this study was to identify potential targets for closed-loop neuromodulation of generalized tonic-clonic seizures. Accordingly, we have tested our hypotheses in a chemical model of generalized convulsive seizures and not genetic models of generalized epilepsy where spike-wave absence seizures are frequently present. Finally, we have analyzed 29 seizures recorded from two rats. Since the focus of this study was to map the dynamics of one seizure subtype (i.e., generalized tonic-clonic seizures), a higher number of subjects are less likely to impact the result.

## 6. Conclusions

In an acute chemical model of generalized tonic-clonic seizure, automated seizure detectors performed better for subcortical structures like thalamus or hippocampus than in cortex. The mean detection latency in thalamus by line length outperformed cortex by 4 seconds. Multiple linear correlations-Granger's Causality revealed a decrease in coupling within the thalamocortical network during generalized tonic-clonic seizures and the decrease in information flow was significant from thalamus to hippocampal-parietal nodes. Overall, the results of this preclinical study indicate that thalamus is a superior target for automated detection of generalized seizures and modulation of synchrony to increase coupling may be a strategy for rational designing of electrical stimulation.

## Figures and Tables

**Table 1:** Classification of the seizures stages using data-driven approach on Granger's Causality data; principle component analysis (PCA) showed that the three stages had distinct mean Granger's Causality values.

G causality mean	Preseizure (eigenvalue)	Seizure (eigenvalue)	Postseizure (eigenvalue)
Thalamus-frontal	0.732	0.731	0.777
Frontal-thalamus	0.72	0.677	0.747
Thalamus-parietal	0.626	0.552	0.552

Parietal-thalamus	0.79	0.766	0.882
Thalamus-hippocampus	0.666	0.72	0.734
Hippocampal-thalamus	0.813	0.83	0.865

Extraction method: principal component analysis			
Rotation method: Varimax with Kaiser Normalization			
PC, principal component			

## References

[1] Blumenfeld H., Westerveld M., Ostroff R. B. (2003). Selective frontal, parietal, and temporal networks in generalized seizures. *NeuroImage*.

[2] Langan Y., Nashef L., Sander J. W. (2005). Case-control study of SUDEP. *Neurology*.

[3] Bergey G. K., Morrell M. J., Mizrahi E. M. (2015). Long-term treatment with responsive brain stimulation in adults with refractory partial seizures. *Neurology*.

[4] Gloor P., Quesney L. F., Zumstein H. (1977). Pathophysiology of generalized penicillin epilepsy in the cat: the role of cortical and subcortical structures. II. Topical application of penicillin to the cerebral cortex and to subcortical structures. *Electroencephalography and Clinical Neurophysiology*.

[5] Quesney L. F., Gloor P., Kratzenberg E., Zumstein H. (1977). Pathophysiology of generalized penicillin epilepsy in the cat: The role of cortical and subcortical structures. I. Systemic application of penicillin. *Electroencephalography and Clinical Neurophysiology*.

[6] Avoli M., Gloor P. (1982). Interaction of cortex and thalamus in spike and wave discharges of feline generalized penicillin epilepsy. *Experimental Neurology*.

[7] Meeren H., van Luijtelaar G., Lopes da Silva F., Coenen A. (2005). Evolving concepts on the pathophysiology of absence seizures: the cortical focus theory. *Archives of Neurology*.

[8] Brevard M. E., Kulkarni P., King J. A., Ferris C. F. (2006). Imaging the neural substrates involved in the genesis of pentylenetetrazol-induced seizures. *Epilepsia*.

[9] Craig C. R., Colasanti B. K. (1988). A study of pentylenetetrazol kindling in rats and mice. *Pharmacology Biochemistry and Behavior*.

[10] Jiruska P., de Curtis M., Jefferys J. G. R., Schevon C. A., Schiff S. J., Schindler K. (2013). Synchronization and desynchronization in epilepsy: controversies and hypotheses. *The Journal of Physiology*.

[11] Sohal V. S., Sun F. T. (2011). Responsive neurostimulation suppresses synchronized cortical rhythms in patients with epilepsy. *Neurosurgery Clinics of North America*.

[12] Avoli M. (2014). Mechanisms of epileptiform synchronization in cortical neuronal networks. *Current Medicinal Chemistry*.

[13] Dominguez L. G., Wennberg R. A., Gaetz W., Cheyne D., Snead O. C., Perez Velazquez J. L. (2005). Enhanced synchrony in epileptiform activity? Local versus distant phase synchronization in generalized seizures. *The Journal of Neuroscience*.

[14] Zhong Y., Lu G., Zhang Z., Jiao Q., Li K., Liu Y. (2011). Altered regional synchronization in epileptic patients with generalized tonic-clonic seizures. *Epilepsy Research*.

[15] Serikawa T., MashimO T., Kuramoro T., Voigt B., Ohno Y., Sasa M. (2015). Advances on genetic rat models of epilepsy. *Journal of Experimental Animal Science*.

[16] Coenen A. M. L., Drinkenburg W. H. I. M., Inoue M., van Luijtelaar E. L. J. M. (1992). Genetic models of absence epilepsy, with emphasis on the WAG/Rij strain of rats. *Epilepsy Research*.

[17] Palombi O., Shin J. W., Watson C., Paxinos G. (2006). Neuroanatomical affiliation visualization-interface system. *Neuroinformatics*.

[18] Schwarz A. J., Danckaert A., Reese T. (2006). A stereotaxic MRI template set for the rat brain with tissue class distribution maps and co-registered anatomical atlas: Application to pharmacological MRI. *NeuroImage*.

[19] Zhang T., Zhou J., Jiang R., Yang H., Carney P. R., Jiang H. (2014). Pre-seizure state identified by diffuse optical tomography. *Scientific Reports*.

[20] Luttjohann A., Fabene P. F., van Luijtelaar G. (2009). A revised Racine's scale for PTZ-induced seizures in rats. *Physiology & behavior*.

[21] Esteller R., Echauz J., Tcheng T. Comparison of line length feature before and after brain electrical stimulation in epileptic patients.

[22] Majumdar K. (2012). Differential operator in seizure detection. *Computers in Biology and Medicine*.

[23] Majumdar K. K., Vardhan P. (2011). Automatic seizure detection in ECoG by differential operator and windowed variance. *IEEE Trans Neural Syst Rehabil Eng*.

[24] Friston K. J. (2011). Functional and effective connectivity: a review. *Brain Connectivity*.

[25] Barnett L., Seth A. K. (2014). The MVGC multivariate Granger causality toolbox: a new approach to Granger-causal inference. *Journal of Neuroscience Methods*.

[26] Hettiarachchi I. T., Mohamed S., Nyhof L., Nahavandi S. An extended multivariate autoregressive framework for EEG-based information flow analysis of a brain network.

[27] Genovese C. R., Lazar N. A., Nichols T. (2002). Thresholding of statistical maps in functional neuroimaging using the false discovery rate. *Neuroimage*.

[28] Hu L., Zhang Z. G., Mouraux A., Iannetti G. D. (2015). Multiple linear regression to estimate time-frequency electrophysiological responses in single trials. *Neuroimage*.

[29] St-Laurent M., McCormick C., Cohn M., Mišić B., Giannoylis I., McAndrews M. P. (2014). Using multivariate data reduction to predict postsurgery memory decline in patients with mesial temporal lobe epilepsy. *Epilepsy & Behavior*.

[30] Schelter B., Winterhalder M., Maiwald T. (2006). Do false predictions of seizures depend on the state of vigilance? A report from two seizure-prediction methods and proposed remedies. *Epilepsia*.

[31] Khamis H., Mohamed A., Simpson S. (2013). Frequency-moment signatures: a method for automated seizure detection from scalp EEG. *Clin Neurophysiol*.

[32] Schad A., Schindler K., Schelter B. (2008). Application of a multivariate seizure detection and prediction method to non-invasive and intracranial long-term EEG recordings. *Clinical Neurophysiology*.

[33] Meeren H. K. M., Pijn J. P. M., Van Luijtelaar E. L. J. M., Coenen A. M. L., Da Silva F. H. L. (2002). Cortical focus drives widespread corticothalamic networks during spontaneous absence seizures in rats. *The Journal of Neuroscience*.

[34] Steriade M., Amzica F., Neckelmann D., Timofeev I. (1998). Spike-wave complexes and fast components of cortically generated seizures. II. Extra- and intracellular patterns. *Journal of Neurophysiology*.

[35] Wagner F. B., Truccolo W., Wang J., Nurmikko A. V. (2015). Spatiotemporal dynamics of optogenetically induced and spontaneous seizure transitions in primary generalized epilepsy. *Journal of Neurophysiology*.

[36] Sorokin J. M., Davidson T. J., Frechette E. (2017). Bidirectional Control of Generalized Epilepsy Networks via Rapid Real-Time Switching of Firing Mode. *Neuron*.

[37] Amor F., Baillet S., Navarro V., Adam C., Martinerie J., Le Van Quyen M. (2009). Cortical local and long-range synchronization interplay in human absence seizure initiation. *NeuroImage*.

[38] Ananda S., Nicholls D. P., Mogul D. J. Modulation of instantaneous synchrony during seizures by deep brain stimulation.

[39] Beverlin Ii B., Netoff T. I. (2012). Dynamic control of modeled tonic-clonic seizure states with closed-loop stimulation. *Front Neural Circuits*.

[40] Hauptmann C., Tass P. A. (2010). Restoration of segregated, physiological neuronal connectivity by desynchronizing stimulation. *Journal of Neural Engineering*.

[41] Popovych O. V., Tass P. A. (2014). Control of abnormal synchronization in neurological disorders. *Front Neurol*.

[42] Lian J., Shuai J., Durand D. M. (2004). Control of phase synchronization of neuronal activity in the rat hippocampus. *Journal of Neural Engineering*.

[43] Pantoja-Jimenez C. R., Magdaleno-Madrigal V. M., Almazan-Alvarado S., Fernandez-Mas R. (2014). Anti-epileptogenic effect of high-frequency stimulation in the thalamic reticular nucleus on PTZ-induced seizures. *Brain Stimulation*.

[44] Zhong X.-L., Lv K.-R., Zhang Q. (2011). Low-frequency stimulation of bilateral anterior nucleus of thalamus inhibits amygdale-kindled seizures in rats. *Brain Research Bulletin*.

